# Differences in Influenza Vaccination Coverage between Adult Immigrants and Italian Citizens at Risk for Influenza-Related Complications: A Cross-Sectional Study

**DOI:** 10.1371/journal.pone.0166517

**Published:** 2016-11-10

**Authors:** Massimo Fabiani, Flavia Riccardo, Anteo Di Napoli, Lidia Gargiulo, Silvia Declich, Alessio Petrelli

**Affiliations:** 1 National Centre for Epidemiology, Surveillance and Health Promotion (CNESPS), Italian National Institute of Health (ISS), Rome, Italy; 2 European Programme for Intervention Epidemiology Training (EPIET), European Centre for Disease Prevention and Control (ECDC), Stockholm, Sweden; 3 National Institute for Health, Migration and Poverty (INMP), Rome, Italy; 4 National Institute of Statistics (ISTAT), Rome, Italy; Australian National University, AUSTRALIA

## Abstract

**Background:**

Due to their increased vulnerability, immigrants are considered a priority group for communicable disease prevention and control in Europe. This study aims to compare influenza vaccination coverage (IVC) between regular immigrants and Italian citizens at risk for its complications and evaluate factors affecting differences.

**Methods:**

Based on data collected by the National Institute of Statistics during a population-based cross-sectional survey conducted in Italy in 2012–2013, we analysed information on 42,048 adult residents (≥ 18 years) at risk for influenza-related complications and with free access to vaccination (elderly residents ≥ 65 years and residents with specific chronic diseases). We compared IVC between 885 regular immigrants and 41,163 Italian citizens using log-binomial models and stratifying immigrants by area of origin and length of stay in Italy (recent: < 10 years; long-term: ≥ 10 years).

**Results:**

IVC among all immigrants was 16.9% compared to 40.2% among Italian citizens (vaccination coverage ratio (VCR) = 0.42, 95% confidence interval (CI): 0.36–0.49). Adjusting for sex, age and area of residence, this difference was greatly reduced but remained statistically significant (VCR = 0.71, 95 CI: 0.61–0.81). Further adjustment for socio-economic factors (education, occupation, family composition and economic status) and a composite indicator of health-services utilization did not affect the difference (VCR = 0.78, 95% CI: 0.68–0.90). However, after adjustments, only long-term immigrants from Africa (VCR = 0.49, 95% CI: 0.28–0.85) and recent immigrants (VCR = 0.58, 95% CI: 0.43–0.78) showed a significantly different IVC compared to Italian citizens.

**Conclusions:**

Differences in demographic characteristics, socio-economic conditions and health-services utilization explained the reduced IVC in most long-term immigrants compared to Italian citizens. By contrast, these differences did not explain the reduced IVC in long-term immigrants from Africa and recent immigrants. This suggests that IVC in these sub-groups is affected by other informal barriers (e.g., cultural and linguistic) that need to be investigated to promote effective immunization access strategies.

## Introduction

The number of international migrants worldwide rapidly increased in the last decades, reaching 244 million in 2015 (3.3% of the world’s population) [1]. Most of them were living in high-income countries (70.8%), where they accounted for 13% of the total population. In January 2015, 35.1 million immigrants (6.9% of the total population) resided in the 28 European Union (EU) countries [2]. Updated estimates from the Italian National Institute of Statistics indicate that the number of foreign citizens legally entitled to stay in Italy (regular immigrants) and formally residing in the country doubled in the last decade, increasing from 2.4 million (4.1% of the resident population) in 2005 to 5.0 million (8.2% of the resident population) in 2015 [3]. Of these, about 3.5 million (70.3%) were citizens of countries outside EU, especially from other European countries (22.7%), Africa (20.5%) and Asia (19.3%) [4].

Although migrants are often relatively healthy on arrival compared with host country populations (the so-called “healthy migrant effect”) [5,6], they tend to become more vulnerable and are therefore considered a priority group for the prevention and control of communicable diseases [7–9]. This increased vulnerability reflects several factors, including, among others, the assimilation to lifestyles associated with disadvantaged socio-economic conditions, cultural characteristics and reduced access to services for health prevention and care [10].

Vaccination is one of the most cost-effective strategies to prevent infectious diseases. The World Health Organization (WHO) reports that licensed vaccines are currently available to prevent, or contribute to the prevention and control of, twenty-five infections [11]. Among these, influenza is an important public health problem, with potentially severe consequences, especially among the elderly and people with chronic diseases [12]. In Italy, seasonal influenza vaccination is recommended and offered free of charge to all residents, including regular immigrants, at risk for influenza-related complications [13]. This group is mostly comprised of elderly people (≥ 65 years of age) and people over six months of age affected by specific chronic conditions. Influenza vaccine for these at risk people is generally provided and administered free of charge by family doctors, who are sensitized to actively promote it.

There is little data available on immunization rates among migrant populations hosted in European countries. Most of this information concerns paediatric vaccinations in immigrant children and adolescents (e.g., measles, mumps and rubella) [14–17], while information on influenza vaccination uptake in adult immigrants is still limited [18,19]. This study aims to compare influenza vaccination coverage (IVC) between adult regular immigrants and Italian citizens at risk for influenza-related complications and evaluate factors affecting differences.

## Materials and Methods

### Study population and data source

In 2012–2013, as part of a multi-purpose surveys system, the Italian National Institute of Statistics conducted a large cross-sectional survey to collect information on health conditions, health determinants and utilization of health services among the general population resident in Italy [20]. This survey was part of the activities included in the 2011–2013 National Statistical Programme (PSN code: IST-02067) approved by the Italian Presidency of the Council of Ministers [21]. The survey unit was the de-facto household, defined as a group of cohabitant individuals linked by marriage, kinship, affinity, adoption, guardianship or affective ties. A total of 60,368 de-facto households were randomly selected within 1456 nationally-representative municipalities. The selected households were informed by letter from the Italian National Institute of Statistics about the purposes and the modalities of conduction of the survey. They were also reassured about confidentiality and protection of personal data. Except for some sensitive information specified in the informative letter, the response to the survey was mandatory by law and formal consent to participate was therefore not required. Out of the sampled households, a total of 49,811 households participated to the survey responding to all questions (response rate = 82.5%). Non-contact (10.7%), due to the absence of the household’s members after repeated home visits (8.4%) and errors in the sampling list (2.3%), was the most frequent reason for non-participation. Refusal to provide non-mandatory sensitive information was the reason for non-participation in 5.7% of the sample. The data collection method did not change during the conduction of the survey and was the same for all participants. Most of information on the 119,073 members of the participating households was collected using face-to-face interviews conducted by specifically trained staff in the household’s residence. The remaining survey questions were answered through self-administered questionnaires. Any personal identifier was removed by the Italian National Institute of Statistics before making data available for this study.

**Fig 1:**
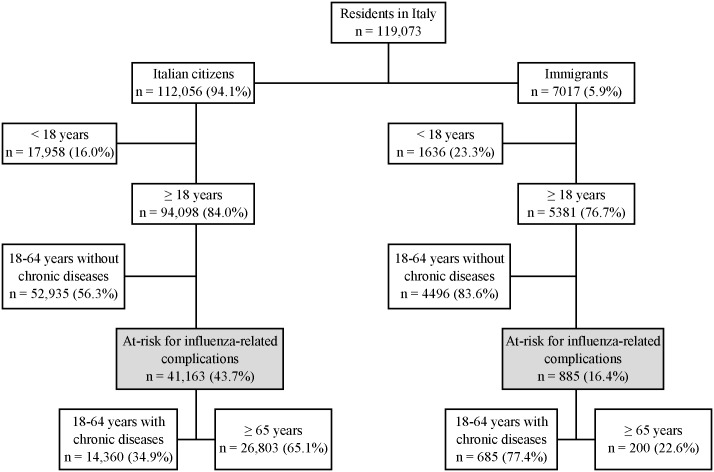
Selection of residents included in the study from the whole sample surveyed by the Italian National Institute of Statistics (Italy, 2012–2013).

From this anonymised dataset, we selected and analysed information on all the 885 regular foreign citizens and 41.163 Italian citizens aged 18 years and over considered at risk for influenza-related complications and with free access to vaccination (Fig 1). These include elderly residents (≥ 65 years of age) and those reporting a diagnosis for at least one of the following chronic conditions: asthma, diabetes, hypertension, myocardial infarction, angina pectoris or other heart diseases, ictus/cerebral haemorrhage, tumour, hepatic cirrhosis, celiac disease, renal insufficiency, and bronchitis/emphysema. We excluded children less than 18 years of age because they accounted for a very small proportion of those targeted for influenza vaccination (2.2%) and information on educational level was not available for this sub-group.

### Exposure, outcome and possible confounders

We assessed the association between citizenship (exposure) and uptake of influenza vaccination in the epidemic season preceding the interview (outcome). Based on recommendations to disaggregate information on migrant health by relevant categories [22], foreign citizens who were regularly resident in Italy (hereafter referred to as “immigrants”) were analysed both as a whole and stratifying by area of origin (based on citizenship) and length of stay in Italy (recent immigrants: < 10 years; long-term immigrants: ≥ 10 years). We considered as possible confounders or mediating factors of the relationship between citizenship and vaccination uptake the demographic characteristics (i.e., age, sex and area of residence) and socio-economic conditions (i.e., educational level, occupational status, household composition, and perception of economic resources), as well as a composite indicator of utilization of health services in the year preceding the interview (i.e., weighted combination of data on hospital admissions, general and specialist medical examinations, diagnostic tests, accesses to local services for rehabilitation, mental health and family planning, and drugs assumption (see S1 Appendix for details)). We also conducted a supplementary analysis taking into account: 1) the elderly separately from adults 18–64 years of age affected by chronic conditions (S1 Table); 2) definitions of recent and long-term immigrants based on 7-year and 13-year thresholds instead of the 10-year threshold used in the main analysis (S2 Table).

### Statistical analysis

We described and compared the demographic and socio-economic characteristics and the composite indicator of utilization of health services between immigrants and Italian citizens using the chi-square test.

Differences in IVC between immigrants and Italian citizens were evaluated using log-binomial models. Vaccination coverage ratios (VCR) and their 95% confidence intervals (CI) were used to describe the strength of the association between citizenship and IVC. In order to assess how much of this association is explained by different characteristics between immigrants and Italian citizens, we first adjusted the analysis by including the demographic variables into the multivariable models. Subsequently, we added the socio-economic variables and then also included the composite indicator of health service utilization. The same hierarchical order of inclusion was used to evaluate, separately for immigrants and Italian citizens, the association between factors other than citizenship and IVC, thus avoiding the over-adjustment for variables that are likely to mediate their effect on vaccination uptake [23]. In particular, we assumed that the demographic characteristics (first level) partly explain the socioeconomic conditions (second level) that, in turn, partly explain the level of utilization of health services (third level). Based on this assumption, only VCR adjusted for factors assigned to the same hierarchical level or the previous ones were presented. In order to evaluate possible differences in the effects of these factors on IVC according to citizenship, we tested the interaction terms between citizenship and each of the other factors included in the multivariable models through the log-likelihood ratio test.

Statistical analysis was performed using Stata/MP version 13 (StataCorp LP, Texas, USA).

## Results

A total of 885 (2.2%) out of the 42,048 adult residents targeted for influenza vaccination were immigrants. They accounted for 16.4% of the 5381 adult immigrants sampled for the survey conducted by the Italian National Institute of Statistics (Fig 1). They were mostly 18-64-years-old adults affected by chronic diseases considered as risk conditions for influenza complications (77.4%). By contrast, out of the 94,098 adult Italian citizens sampled, those targeted for influenza vaccination accounted for 43.7% and were mostly elderly people (65.1%).

### Demographic and socio-economic characteristics and health services utilization

Among residents targeted for influenza vaccination, immigrants showed a significantly different demographic and socio-economic profile compared with Italian citizens (Table 1). Immigrants were younger than Italian citizens (36.0% *vs* 7.8% were less than 45 years of age; p < 0.001) and had a higher level of education (42.8% *vs* 24.9% completed high school; p < 0.001) and rate of employment (45.2% *vs* 19.2%; p < 0.001). They reported more frequently than Italian citizens to live in large households (62.1% *vs* 40.5%; p < 0.001), without adequate economic resources (59.1% *vs* 38.9%; p < 0.001), and to experience a reduced utilization of health services (p < 0.001).

**Table 1:** Demographic and socio-economic characteristics of adult Italian citizens and immigrants at risk for influenza-related complications (Italy, 2012–2013).*

	Italian citizens and all immigrants			Immigrants by length of stay			Immigrants by area of origin					
	Italian citizens	Immigrants	p-value	< 10 years	≥ 10 years	p-value	West Europe	East Europe	Africa	Asia and Oceania	America	p-value
	n (%)	n (%)		n (%)	n (%)		n (%)	n (%)	n (%)	n (%)	n (%)	
**Sex**			< 0.001			0.001						< 0.001
Male	18,689 (45.4)	319 (36.1)		88 (28.8)	231 (39.9)		29 (35.4)	136 (31.3)	86 (52.8)	46 (45.1)	22 (21.2)	
Female	22,474 (54.6)	566 (63.9)		218 (71.2)	348 (60.1)		53 (64.6)	298 (68.7)	77 (47.2)	56 (54.9)	82 (78.8)	
**Age group**			< 0.001			< 0.001						< 0.001
18–24 years	465 (1.1)	31 (3.5)		21 (6.9)	10 (1.7)		2 (2.4)	15 (3.5)	4 (2.4)	4 (3.9)	6 (5.8)	
25–44 years	2759 (6.7)	288 (32.5)		118 (38.5)	170 (29.4)		7 (8.5)	143 (32.9)	65 (39.9)	39 (38.2)	34 (32.7)	
45–64 years	11,136 (27.1)	366 (41.4)		108 (35.3)	258 (44.6)		19 (23.2)	205 (47.2)	57 (35.0)	45 (44.1)	40 (38.5)	
65+ years	26,803 (65.1)	200 (22.6)		59 (19.3)	141 (24.3)		54 (65.9)	71 (16.4)	37 (22.7)	14 (13.7)	24 (23.1)	
**Area of residence**			< 0.001			0.018						< 0.001
North-west	9425 (22.9)	274 (31.0)		93 (30.4)	181 (31.3)		19 (23.2)	107 (24.7)	69 (42.3)	37 (36.3)	42 (40.4)	
North-east	8401 (20.4)	280 (31.6)		97 (31.7)	183 (31.6)		22 (26.8)	148 (34.1)	52 (31.9)	37 (36.3)	21 (20.2)	
Centre	7547 (18.3)	198 (22.4)		56 (18.3)	142 (24.5)		23 (28.1)	106 (24.4)	21 (12.9)	19 (18.6)	29 (27.9)	
South and islands	15,790 (38.4)	133 (15.0)		60 (19.6)	73 (12.6)		18 (21.9)	73 (16.8)	21 (12.9)	9 (8.8)	12 (11.5)	
**Educational level**			< 0.001			0.333						< 0.001
High (≥ 13 years)	10,251 (24.9)	379 (42.8)		129 (42.2)	250 (43.2)		51 (62.2)	200 (46.1)	35 (21.5)	36 (35.3)	57 (54.8)	
Medium (8–12 years)	12,022 (29.2)	316 (35.7)		103 (33.6)	213 (36.8)		24 (29.3)	166 (38.2)	49 (30.1)	44 (43.1)	33 (31.7)	
Low (< 8 years)	18,890 (45.9)	190 (21.5)		74 (24.2)	116 (20.0)		7 (8.5)	68 (15.7)	79 (48.5)	22 (21.6)	14 (13.5)	
**Occupational status**			< 0.001			0.108						< 0.001
Employed	7911 (19.2)	400 (45.2)		127 (41.5)	273 (47.1)		18 (21.9)	219 (50.5)	58 (35.6)	56 (54.9)	49 (47.1)	
Unemployed	33,252 (80.8)	485 (54.8)		179 (58.5)	306 (52.9)		64 (78.1)	215 (49.5)	105 (64.4)	46 (45.1)	55 (52.9)	
**Household composition**			< 0.001			0.443						< 0.001
Person living alone	9343 (22.7)	194 (21.9)		67 (21.9)	127 (21.9)		33 (40.2)	104 (24.0)	19 (11.7)	16 (15.7)	22 (21.1)	
Couple without children	15,147 (36.8)	142 (16.0)		55 (18.0)	87 (15.0)		23 (28.1)	71 (16.4)	15 (9.2)	14 (13.7)	19 (18.3)	
Couple with children	11,687 (28.4)	373 (42.2)		131 (42.8)	242 (41.8)		15 (18.3)	167 (38.5)	99 (60.7)	51 (50.0)	41 (39.4)	
Other families	4986 (12.1)	176 (19.9)		53 (17.3)	123 (21.3)		11 (13.4)	92 (21.2)	30 (18.4)	21 (20.6)	22 (22.1)	
**Economic resources**			< 0.001			0.144						< 0.001
Adequate	25,133 (61.1)	362 (40.9)		115 (37.6)	247 (42.7)		66 (80.5)	169 (38.9)	34 (20.9)	41 (40.2)	52 (50.0)	
Non adequate	16,030 (38.9)	523 (59.1)		191 (62.4)	332 (57.3)		16 (19.5)	265 (61.1)	129 (79.1)	61 (59.8)	52 (50.0)	
Use of health services**			< 0.001			0.206						0.126
1st quintile	8132 (19.8)	297 (33.6)		115 (37.6)	182 (31.4)		19 (23.2)	145 (33.4)	65 (39.9)	30 (29.4)	38 (36.5)	
2nd quintile	12,185 (29.5)	255 (28.8)		90 (29.4)	165 (28.5)		28 (34.1)	126 (29.0)	42 (25.8)	34 (33.3)	25 (24.0)	
3rd quintile	4319 (10.5)	78 (8.8)		22 (7.2)	56 (9.7)		5 (6.1)	41 (9.5)	13 (8.0)	12 (11.8)	7 (6.7)	
4th quintile	8307 (20.2)	138 (15.6)		46 (15.0)	92 (15.9)		15 (18.3)	72 (16.6)	27 (16.6)	12 (11.8)	12 (11.5)	
5th quintile	8220 (20.0)	117 (13.2)		33 (10.8)	84 (14.5)		15 (18.3)	50 (11.5)	16 (9.8)	14 (13.7)	22 (21.2)	

* People considered at risk for influenza-related complications were the elderly (65+ years) and people with a diagnosis for at least one of the following chronic conditions: asthma, diabetes, hypertension, myocardial infarction, angina pectoris or other heart diseases, ictus/cerebral haemorrhage, tumour, hepatic cirrhosis, celiac disease, renal insufficiency, and bronchitis/emphysema.

** Relative composite indicator (range: 0–100) based on a weighted combination of data on hospital admissions (duration of hospital stay and occurrence of surgical intervention), number of general and specialist medical examinations and diagnostic tests, number of accesses to local services for rehabilitation, mental health and family planning, and frequency of drugs assumption in the year preceding the interview (see S1 Appendix for details).

A total of 306 (34.6%) immigrants were resident in Italy since less than ten years. Compared with long-term immigrants, recent immigrants were younger (45.4% *vs* 31.1% were less than 45 years of age; p < 0.001) and had a higher proportion of females (71.2% *vs* 60.1%; p = 0.001) (Table 1). Moreover, they reported more frequently to live in southern Italy (19.6% *vs* 12.6%). Overall, most of immigrants were from eastern Europe (n = 434, 49.0%) and Africa (n = 163, 18.4%). Immigrants showed a significantly different demographic and socio-economic profile according to the geographical area of origin (Table 1). In particular, immigrants from Africa reported worse socio-economic conditions and a reduced utilization of health services compared to other immigrants, especially those from western Europe.

### Influenza vaccination coverage

**Fig 2:**
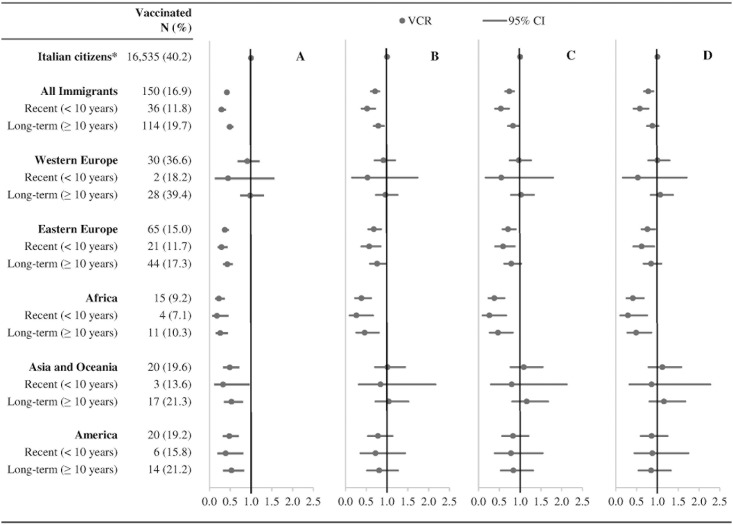
Influenza vaccination coverage among adult Italian citizens and immigrants at risk for influenza-related complications (Italy, 2012–2013). VCR, vaccination coverage ratio; CI, confidence interval. * Reference category for all VCRs. (A) Crude VCR. (B) VCR adjusted for sex, age and area of residence. (C) VCR adjusted for sex, age, area of residence, educational level, occupational status, household composition, and economic resources. (D) VCR adjusted for sex, age, area of residence, educational level, occupational status, household composition, economic resources, and health services utilization index.

Overall, IVC among residents at risk for influenza-related complications was 39.7%; 16.9% among immigrants compared to 40.2% among Italian citizens (VCR = 0.42, 95% CI: 0.36–0.49) (Fig 2A). Adjusting for demographic characteristics (i.e., sex, age and area of residence), this difference was greatly reduced but remained statistically significant (VCR = 0.71, 95% CI: 0.61–0.81) (Fig 2B). Additional adjustment for socio-economic factors (i.e., educational level, occupational status, household composition, and perception of economic resources) did not affect the difference (VCR = 0.74, 95% CI: 0.64–0.85) (Fig 2C), as well as further adjustment for the composite indicator of health-services utilization (VCR = 0.78, 95% CI: 0.68–0.90) (Fig 2D).

After adjustments, recent immigrants showed a significantly different IVC compared to Italian citizens (VCR = 0.58, 95% CI: 0.43–0.78; p < 0.001), especially those from Africa (VCR = 0.29, 95% CI: 0.11–0.75) and eastern Europe (VCR = 0.62, 95% CI: 0.42–0.92) (Fig 2D). We also observed a reduced IVC among long-term immigrants from Africa (VCR = 0.49, 95% CI: 0.28–0.85), while long-term immigrants from other areas did not show significant differences. The supplementary analysis showed how the reduced IVC in both short-term and long-term immigrants from Africa compared to Italian citizens was more pronounced in the elderly than in adults affected by chronic conditions (S1 Table). Moreover, it showed how the results did not substantially change when the 10-year length of stay threshold defining recent and long-term immigrants was replaced by 7-year and 13-year thresholds (S2 Table).

The analysis of factors associated with IVC is presented in Table 2 separately for Italian citizens and immigrants. The two groups showed a similar profile, except for the educational level (log-likelihood interaction test, P = 0.033). Low education, as compared to high education, was associated with an increased vaccination uptake among Italian citizens (VCR = 1.16, 95% CI: 1.12–1.20), but not among immigrants (VCR = 0.84, 95% CI: 0.56–1.25). In both groups, IVC tended to be associated with increased age, unemployment, living alone or just with a partner, and increased utilization of health services. No relevant differences in IVC were observed according to sex and perceived adequacy of economic resources in both Italian citizens and immigrants.

**Table 2:** Factors associated with influenza vaccination among adult Italian citizens and immigrants at risk for influenza-related complications (Italy, 2012–2013).

	Italian citizens			Immigrants		
	Unvaccinated	Vaccinated	VCR^a^	Unvaccinated	Vaccinated	VCR^a^
	n (%)	n (%)	(95% CI)	n (%)	n (%)	(95% CI)
**Sex (1)**						
Male	11,345 (60.7)	7344 (39.3)	1	268 (84.0)	51 (16.0)	1
Female	13,283 (59.1)	9191 (40.9)	1.00 (0.98–1.02)	467 (82.5)	99 (17.5)	1.02 (0.76–1.37)
**Age group (1)**						
18–24 years	416 (89.5)	49 (10.5)	1	28 (90.3)	3 (9.7)	1
25–44 years	2467 (89.4)	292 (10.6)	1.01 (0.76–1.34)	272 (94.4)	16 (5.6)	0.59 (0.18–1.92)
45–64 years	8888 (79.8)	2248 (20.2)	1.92 (1.47–2.51)	307 (83.9)	59 (16.1)	1.67 (0.56–5.01)
65+ years	12,857 (48.0)	13,946 (52.0)	4.95 (3.79–6.45)	128 (64.0)	72 (36.0)	3.66 (1.23–10.9)
**Area of residence (1)**						
North-west	5906 (62.7)	3519 (37.3)	1	244 (89.0)	30 (11.0)	1
North-east	4846 (57.7)	3555 (42.3)	1.14 (1.10–1.17)	223 (79.6)	57 (20.4)	1.65 (1.12–2.45)
Centre	4356 (57.7)	3191 (42.3)	1.12 (1.08–1.16)	157 (79.3)	41 (20.7)	1.48 (0.98–2.26)
South and islands	9520 (60.3)	6270 (39.7)	1.08 (1.05–1.11)	111 (83.5)	22 (16.5)	1.29 (0.79–2.11)
Educational level (2)*						
High (≥ 13 years)	7309 (71.3)	2942 (28.7)	1	319 (84.2)	60 (15.8)	1
Medium (8–12 years)	8168 (67.9)	3854 (32.1)	1.00 (0.96–1.04)	263 (83.2)	53 (16.8)	1.19 (0.87–1.64)
Low (< 8 years)	9151 (48.4)	9739 (51.6)	1.16 (1.12–1.20)	153 (80.5)	37 (19.5)	0.84 (0.56–1.25)
**Occupational status (2)**						
Employed	6659 (84.2)	1252 (15.8)	1	359 (89.8)	41 (10.2)	1
Unemployed	17,969 (54.0)	15,283 (46.0)	1.45 (1.37–1.54)	376 (77.5)	109 (22.5)	1.54 (1.07–2.24)
**Household composition (2)**						
Person living alone	4758 (50.9)	4585 (49.1)	1	152 (78.3)	42 (21.7)	1
Couple without children	7965 (52.6)	7182 (47.4)	0.98 (0.95–1.00)	111 (78.2)	31 (21.8)	0.90 (0.61–1.33)
Couple with children	8827 (75.5)	2860 (24.5)	0.79 (0.76–0.82)	328 (87.9)	45 (12.1)	0.71 (0.48–1.05)
Other families	3078 (61.7)	1908 (38.3)	0.90 (0.86–0.93)	144 (81.8)	32 (18.2)	0.82 (0.55–1.22)
**Economic resources (2)**						
Adequate	15,119 (60.2)	10,014 (39.8)	1	291 (80.4)	71 (19.6)	1
Non adequate	9509 (59.3)	6521 (40.7)	1.00 (0.98–1.03)	444 (84.9)	79 (15.1)	0.93 (0.69–1.25)
**Use of health services (3)**						
1st quintile	6158 (75.7)	1974 (24.3)	1	273 (91.9)	24 (8.1)	1
2nd quintile	7507 (61.6)	4678 (38.4)	1.51 (1.44–1.57)	205 (80.4)	50 (19.6)	1.85 (1.19–2.88)
3rd quintile	2367 (54.8)	1952 (45.2)	1.64 (1.57–1.72)	60 (76.9)	18 (23.1)	2.24 (1.32–3.82)
4th quintile	4314 (51.9)	3993 (48.1)	1.74 (1.67–1.82)	104 (75.4)	34 (24.6)	2.59 (1.64–4.09)
5th quintile	4282 (52.1)	3938 (47.9)	1.71 (1.64–1.78)	93 (79.5)	24 (20.5)	2.45 (1.47–4.08)

VCR, vaccination coverage ratio; CI, confidence interval.

Numbers in parentheses near the variable names indicate the hierarchical level assigned to each factor in multivariable analysis (from 1 to 3).

^a^ VCR adjusted for all the factors assigned to the same hierarchical level or the previous ones.

* Statistically significant interaction with citizenship according to log-likelihood ratio test (P < 0.05).

## Discussion

This study showed a reduced IVC among immigrants compared with Italian citizens. This difference was particularly evident among African immigrants and among recent immigrants, regardless of area of origin. This result is in line with general findings from Spain, where IVC among immigrants was found to be 22.0% compared to 50.4% among nationals [18], and those from another study conducted in Italy, where, among adults less than 65 years of age at risk for influenza-related complications, it was observed a reduced IVC in immigrants compared to Italian citizens (17% *vs* 26%) [19]. Our results are also consistent with findings from the few other studies conducted in Europe that compared immunization rates for infections other than influenza between nationals and migrants [14–17]. All of these studies focussed on paediatric vaccinations (e.g., measles, mumps and rubella), showing reduced immunization rates in immigrant children and adolescents compared with nationals.

Differences in demographic and socio-economic profile and the general utilization of health services only partially explained the reduced IVC in all immigrants compared to Italian citizens. In particular, we found that, after adjustments, this residual unexplained difference was still significant for all recent immigrants and long-term immigrants from Africa. For other long-term immigrants this difference was mainly reduced by adjusting for demographic factors. These findings suggest that the reinforcement of influenza immunization promoting strategies targeting young adults with chronic diseases might be effective in reducing differences in IVC between Italian citizens and most at risk long-term immigrants. However, they also suggest that this approach may not be enough to reduce these differences among recent immigrants and among immigrants from Africa, regardless of their length of stay. Given the equivalent entitlement to free immunization for all regular immigrants in Italy, it is likely that informal barriers are disproportionally hindering influenza vaccination uptake in these two sub-groups. Cultural and linguistic barriers might be playing a predominant role. There are known challenges for immigrants to access routine vaccination services. Immigrants could be unaware of these services due to linguistic difficulties, be unaware they are entitled to access them free of charge, or they could be unwilling to use them for cultural, religious or other reasons [7]. The provision of culturally sensitive information in the community languages, training of professionals and services tailored to the specific needs of immigrants, and the identification and training of key individuals from the migrant community to inform and motivate immigrants to get vaccinated are among the proposed measures to overcome informal barriers to immunization [24–27].

We observed no differences between immigrants and Italian citizens in reference to factors associated with IVC, except for education, which was not associated with vaccination uptake among immigrants but negatively associated with it among Italian citizens. Consistently with findings from another study conducted in Italy [19], influenza vaccination uptake in the whole population was associated with increased age, lower education, unemployment and non-adequate economic resources. Our results are also consistent with findings from a Spanish study showing that primary vaccination coverage was relatively higher in indigenous children than in immigrant children among those whose parents had less than 12 years of education. [15]. Finally, data from Germany on paediatric vaccinations for measles, mumps and rubella [16,28] support the association between increased IVC and low educational level and socio-economic status that we observed in the whole population (immigrants and Italian citizens combined).

Overall, IVC among residents at risk for influenza complications was 39.7%, a rate quite far from the target of 75% recommended by the Council of the European Union [29]. It was also lower than the IVC estimate derived from the same survey conducted in 2005 by the Italian National Institute of Statistics (48.7%), this trend probably reflecting, as for other vaccines, the recent increase in vaccine hesitancy related to several factors (e.g., concerns about vaccine safety and efficacy) [30,31]. While IVC among elderly residents was higher than the median value observed in 24 European countries (51.9% *vs* 44.7%), it was much lower among adult residents less than 65 years of age affected by chronic conditions (17.7% *vs* 45.6% in 7 EU countries with information available) [32].

In our analysis we used citizenship to define immigrant status. In Italy, citizenship rules are based on *ius sanguinis*. Offspring of immigrants that was born and raised in Italy can acquire Italian citizenship at 18 years of age. In our sample, including only adults aged 18 years or more, they are therefore classified as Italian citizens in most cases (only 13 people born in Italy were foreign citizens and 9 of them were citizens of developed countries in western Europe). However, it is worthwhile to note that immigration to Italy is a quite recent phenomenon that significantly increased only from the year 2000. The number of adult second-generation immigrants in Italy is therefore still very limited.

The study sample did not include irregular migrants and those regularly present in Italy but without formal residence in the country. IVC in these migrant groups, who were estimated to account for about 6% and 7% of all migrants, respectively [4,33], is likely to be lower than that among regular resident immigrants. In fact, although legally entitled to anonymously benefit from health preventive services, they are probably less likely to be aware of their right to access health services, might be concerned of the cost of services, or might be afraid to be identified by national authorities [10].

Moreover, this study did not assess IVC among risk categories other than the elderly and people affected by specific chronic conditions. In Italy, as in other European countries, seasonal influenza vaccination is also recommended to other population groups such as pregnant women in their 2^nd^ or 3^rd^ trimester of gestation, healthcare workers and other occupational groups, household contacts of people at risk for influenza-related complications, and residents of long-stay care facilities [13]. However, these additional groups account for a small proportion of those targeted for influenza vaccination and their exclusion is unlikely to have introduced a selection bias in IVC estimates [34].

Finally, our study presented estimates based on self-reported influenza vaccination uptake and diagnoses of chronic diseases that might be affected by recall and social desirability biases [35,36]. This could have led to overestimate IVC, especially among immigrants [36], thus masking an even greater difference in IVC between immigrants and Italian citizens.

Despite the limitations described above, this study is based on a sample size large enough to detect relevant differences as statistically significant with an adequate statistical power, although it was reduced when comparing IVC between Italian citizens and small sub-groups of immigrants.

Moreover, the analysis was conducted on all the adult residents at risk for influenza-related complications included in a sample of the general population resident in Italy. The demographic characteristics of this sample (i.e., distribution of sex, age and area of residence by citizenship) were consistent with national figures at the time the survey was conducted [3]. We also found the educational level by citizenship to be consistent with national figures derived from the 2011 census data [37]. This suggests that the whole sample and the sub-group analysed in this study were representative of the respective country’s reference populations.

Finally, although our study was focusing on IVC rather than incidence of influenza, the analysis was conducted according to a multidimensional framework which was shown to be well suited to describe the complexity and concurrence of risk factors associated with infectious diseases in migrant populations [38].

## Conclusions

Differences in demographic characteristics, socio-economic conditions and health service utilization were found to explain the reduced IVC in most long-term immigrants compared to Italian citizens. By contrast, these differences did not explain the reduced IVC in long-term immigrants from Africa and recent immigrants. Other informal barriers, such as cultural and linguistic barriers, are likely to play an important role among these sub-groups of immigrants. Further investigations, based on both qualitative and quantitative research, are needed to identify which factors are affecting influenza vaccination uptake in these vulnerable at risk sub-groups. This would allow to adequately plan effective strategies to increase influenza vaccination uptake, such as the development of culturally competent health services.

## Supporting Information

S1 Appendix Calculation of the relative composite indicator of health-services utilization. (PDF)

S1 Table Influenza vaccination coverage among Italian citizens and immigrants by category of risk for influenza-related complications (Italy, 2012–2013). (DOCX)

S2 Table Influenza vaccination coverage among Italian citizens and immigrants classified as recent/long-term according to different thresholds (Italy, 2012–2013). (DOCX)
