# Supplementary material for: Differences in Influenza Vaccination Coverage between Adult Immigrants and Italian Citizens at Risk for Influenza-Related Complications: A Cross-Sectional Study
Source: PLoS One. 2016 Nov 10;11(11):e0166517. doi: 10.1371/journal.pone.0166517 (PMC5104396; doi:10.1371/journal.pone.0166517)
Supplement: S1 Appendix — (PDF) [file pone.0166517.s001.pdf]

S1 Appendix

Calculation of the relative composite indicator of health-services utilization

The relative composite indicator of health-services utilization was built combining for each individual different information about the utilization of health services in periods preceding the interview. To account for the relative importance of the different health services, weights proportional to their economic costs were used.
The reference period of information about health-services utilization varied according to the type of service. For this reason data were re-proportioned using coefficients to make utilization of different health-services referable to the same time period (1 year).
The relative composite indicator (range: 0-100) was obtained by dividing the absolute composite indicator by its maximum and multiplying it by 100.

Table. Health-services and factors included in the calculation of I-HSU

Health services (j)		Reference period (t_j)	Frequency (f_{ij})	Weight (ω_j)
Hospital admissions	Medical treatment	3 months	Length of stay (days)	15
	Surgical intervention during last admission in the last 3 months	12 months[a]	Yes=1/No=0	10
General medical examinations		1 month	No. of visits	0.5
Specialist medical examinations and diagnostic tests	Specialist examinations	1 month	No. of visits	1
	Blood tests		No. of tests	0.5
	Urine test			0.15
	Other diagnostic tests			1
Local health services	Rehabilitation	3 months	No. of accesses	1
	Family planning			
	Mental health			
Drugs	Regular assumption	12 months	No. of assumption days[b]	0.05*(1.3 if multiple assumption)[c]
	In the last 2 weeks	12 months[a]	Yes=1/No=0	0.3

[a] A reference period of 12 months was used for the purpose of calculation.
[b] The number of assumption days was derived as follows: never or don't know = 0 days; less than sometimes a week = 15 days; sometimes a week = 104 days; daily = 365 days.
[c] Multiple assumption is defined as regular assumption of at least two different drugs or assumption of at least three different drugs for at least one month. In case of multiple assumption, a supplementary multiplicative weight equal to 1.3 is applied.

For each individual (i), the relative composite indicator of health-services utilization ($I\text{-}HSU_i$) was calculated as follows:

$$\mathbf{I\text{-}HSU_i} = \left[\sum_j \left(f_{ij} \times \omega_j \times 12/t_j\right)\right] / Max_i\left\{\left[\sum_j \left(f_{ij} \times \omega_j \times 12/t_j\right)\right]\right\} * 100$$

This document was prepared in English by the authors on the basis of the methodological notes released by the Italian National Institute of Statistics and described in Italian in:

Gargiulo L, Iannucci L, Quattrociocchi G, Sebastiani A, Tinto A. Innovazioni di processo nell'indagine ISTAT sulla salute. I quaderni di monitor. 2008; 22(suppl 3): 16-28. Available: http://www.agenas.it/images/agenas/monitor/quaderno/pdf/5__ISTAT_salute_2005.pdf
