# Supplementary material for: Differences in Influenza Vaccination Coverage between Adult Immigrants and Italian Citizens at Risk for Influenza-Related Complications: A Cross-Sectional Study
Source: PLoS One. 2016 Nov 10;11(11):e0166517. doi: 10.1371/journal.pone.0166517 (PMC5104396; doi:10.1371/journal.pone.0166517)
Supplement: S1 Table — (DOCX) [file pone.0166517.s002.docx]

**S1 Table**. Influenza vaccination coverage among Italian citizens and immigrants by category of risk for influenza-related complications (Italy, 2012-2013).

|  | **Elderly (65+ years)** | | |  | **Adults affected by chronic diseases* (18-64 years)** | | |
| --- | --- | --- | --- | --- | --- | --- | --- |
|  | **Unvaccinated**  **N (%)** | **Vaccinated**  **N (%)** | **VCR^a^**  **(95% CI)** |  | **Unvaccinated**  **N (%)** | **Vaccinated**  **N (%)** | **VCR^a^**  **(95% CI)** |
| **Italian citizens**** | 12,857 (48.0) | 13,946 (52.0) | 1 |  | 11,771 (82.0) | 2589 (18.0) | 1 |
|  |  |  |  |  |  |  |  |
| **Immigrants** | 128 (64.0) | 72 (36.0) | 0.81 (0.68-0.97) |  | 607 (88.6) | 78 (11.4) | 0.79 (0.64-0.98) |
|  |  |  |  |  |  |  |  |
| Length of stay < 5 years | 46 (78.0) | 13 (22.0) | 0.51 (0.32-0.82) |  | 224 (90.7) | 23 (9.3) | 0.67 (0.45-0.99) |
| Length of stay ≥ 5 years | 82 (58.2) | 59 (41.8) | 0.92 (0.77-1.11) |  | 383 (87.4) | 55 (12.6) | 0.85 (0.67-1.09) |
|  |  |  |  |  |  |  |  |
| **West Europe** | 27 (50.0) | 27 (50.0) | 1.04 (0.81-1.34) |  | 25 (89.3) | 3 (10.7) | 0.66 (0.22-1.92) |
| Length of stay < 5 years | 4 (66.7) | 2 (33.3) | 0.63 (0.21-1.92) |  | 5 (100.0) | 0 (0.0) | undefined^b^ |
| Length of stay ≥ 5 years | 23 (47.9) | 25 (52.1) | 1.10 (0.86-1.42) |  | 20 (87.0) | 3 (13.0) | 0.77 (0.27-2.24) |
|  |  |  |  |  |  |  |  |
| **East Europe** | 45 (63.4) | 26 (36.6) | 0.85 (0.64-1.15) |  | 324 (89.3) | 39 (10.7) | 0.73 (0.54-0.99) |
| Length of stay < 5 years | 18 (75.0) | 6 (25.0) | 0.58 (0.29-1.16) |  | 140 (90.3) | 15 (9.7) | 0.67 (0.41-1.08) |
| Length of stay ≥ 5 years | 27 (57.4) | 20 (42.6) | 0.99 (0.73-1.36) |  | 184 (88.5) | 24 (11.5) | 0.78 (0.54-1.13) |
|  |  |  |  |  |  |  |  |
| **Africa** | 34 (91.9) | 3 (8.1) | 0.19 (0.06-0.55) |  | 114 (90.5) | 12 (9.5) | 0.66 (0.39-1.13) |
| Length of stay < 5 years | 18 (94.7) | 1 (5.3) | 0.12 (0.02-0.82) |  | 34 (91.9) | 3 (8.1) | 0.62 (0.22-1.80) |
| Length of stay ≥ 5 years | 16 (88.9) | 2 (11.1) | 0.26 (0.07-0.95) |  | 80 (89.9) | 9 (10.1) | 0.66 (0.36-1.23) |
|  |  |  |  |  |  |  |  |
| **Asia and Oceania** | 6 (42.9) | 8 (57.1) | 1.35 (0.90-2.00) |  | 76 (86.4) | 12 (13.6) | 0.97 (0.58-1.62) |
| Length of stay < 5 years | 1 (50.0) | 1 (50.0) | 1.51 (0.53-4.24) |  | 18 (90.0) | 2 (10.0) | 0.73 (0.20-2.58) |
| Length of stay ≥ 5 years | 5 (41.7) | 7 (58.3) | 1.33 (0.87-2.04) |  | 58 (85.3) | 10 (14.7) | 1.04 (0.60-1.81) |
|  |  |  |  |  |  |  |  |
| **America** | 16 (66.7) | 8 (33.3) | 0.72 (0.41-1.25) |  | 68 (85.0) | 12 (15.0) | 1.19 (0.73-1.96) |
| Length of stay < 5 years | 5 (62.5) | 3 (37.5) | 1.04 (0.46-2.36) |  | 27 (90.0) | 3 (10.0) | 0.82 (0.28-2.36) |
| Length of stay ≥ 5 years | 11 (68.8) | 5 (31.2) | 0.61 (0.30-1.26) |  | 41 (82.0) | 9 (18.0) | 1.39 (0.80-2.41) |

VCR, vaccination coverage ratio; CI, confidence interval.

* Adults with a diagnosis for at least one of the following chronic conditions: asthma, diabetes, hypertension, myocardial infarction, angina pectoris or other heart diseases, ictus/cerebral haemorrhage, tumour, hepatic cirrhosis, celiac disease, renal insufficiency, and bronchitis/emphysema.

** Reference category for all VCRs presented in the table.

^a^ VCR adjusted for sex, age, area of residence, educational level, occupational status, household composition, economic resources, and health services utilization index.

^b^ No cases in the exposure group.
