# Supplementary material for: Differences in Influenza Vaccination Coverage between Adult Immigrants and Italian Citizens at Risk for Influenza-Related Complications: A Cross-Sectional Study
Source: PLoS One. 2016 Nov 10;11(11):e0166517. doi: 10.1371/journal.pone.0166517 (PMC5104396; doi:10.1371/journal.pone.0166517)
Supplement: S2 Table — (DOCX) [file pone.0166517.s003.docx]

**S2 Table**. Influenza vaccination coverage among Italian citizens and immigrants classified as recent/long-term according to different thresholds (Italy, 2012-2013).

|  | **Threshold: 7 years^1^** | | |  | **Threshold: 13 years^2^** | | |
| --- | --- | --- | --- | --- | --- | --- | --- |
|  | **Unvaccinated**  **N (%)** | **Vaccinated**  **N (%)** | **VCR^a^**  **(95% CI)** |  | **Unvaccinated**  **N (%)** | **Vaccinated**  **N (%)** | **VCR^a^**  **(95% CI)** |
| **Italian citizens*** | 24,628 (59.8) | 16,535 (40.2) | 1 |  | 24,628 (59.8) | 16,535 (40.2) | 1 |
|  |  |  |  |  |  |  |  |
| **Immigrants** | 735 (83.1) | 150 (16.9) | 0.78 (0.68-0.90) |  | 735 (83.1) | 150 (16.9) | 0.78 (0.68-0.90) |
|  |  |  |  |  |  |  |  |
| Recent | 158 (90.3) | 17 (9.7) | 0.45 (0.29-0.70) |  | 449 (85.7) | 75 (14.3) | 0.68 (0.56-0.83) |
| Long-term | 577 (81.3) | 133 (18.7) | 0.87 (0.75-1.00) |  | 286 (79.2) | 75 (20.8) | 0.92 (0.76-1.10) |
|  |  |  |  |  |  |  |  |
| **West Europe** | 52 (63.4) | 30 (36.6) | 1.00 (0.78-1.29) |  | 52 (63.4) | 30 (36.6) | 1.00 (0.78-1.29) |
| Recent | 6 (85.7) | 1 (14.3) | 0.37 (0.06-2.28) |  | 18 (69.2) | 8 (30.8) | 0.86 (0.51-1.43) |
| Long-term | 46 (61.3) | 29 (38.7) | 1.07 (0.84-1.36) |  | 34 (60.7) | 22 (39.3) | 1.07 (0.80-1.41) |
|  |  |  |  |  |  |  |  |
| **East Europe** | 369 (85.0) | 65 (15.0) | 0.76 (0.62-0.94) |  | 369 (85.0) | 65 (15.0) | 0.76 (0.62-0.94) |
| Recent | 85 (91.4) | 8 (8.6) | 0.45 (0.23-0.86) |  | 265 (86.6) | 41 (13.4) | 0.67 (0.51-0.89) |
| Long-term | 284 (83.3) | 57 (16.7) | 0.85 (0.68-1.06) |  | 104 (81.3) | 24 (18.7) | 1.00 (0.73-1.37) |
|  |  |  |  |  |  |  |  |
| **Africa** | 148 (90.8) | 15 (9.2) | 0.41 (0.26-0.67) |  | 148 (90.8) | 15 (9.2) | 0.41 (0.26-0.67) |
| Recent | 37 (92.5) | 3 (7.5) | 0.27 (0.09-0.81) |  | 75 (90.4) | 8 (9.6) | 0.42 (0.22-0.81) |
| Long-term | 111 (90.2) | 12 (9.8) | 0.48 (0.28-0.81) |  | 73 (91.2) | 7 (8.8) | 0.40 (0.20-0.82) |
|  |  |  |  |  |  |  |  |
| **Asia and Oceania** | 82 (80.4) | 20 (19.6) | 1.12 (0.79-1.57) |  | 82 (80.4) | 20 (19.6) | 1.12 (0.79-1.57) |
| Recent | 12 (85.7) | 2 (14.3) | 0.84 (0.26-2.69) |  | 36 (85.7) | 6 (14.3) | 0.80 (0.40-1.61) |
| Long-term | 70 (79.5) | 18 (20.5) | 1.15 (0.81-1.64) |  | 46 (76.7) | 14 (23.3) | 1.38 (0.98-1.96) |
|  |  |  |  |  |  |  |  |
| **America** | 84 (80.8) | 20 (19.2) | 0.86 (0.60-1.24) |  | 84 (80.8) | 20 (19.2) | 0.86 (0.60-1.24) |
| Recent | 18 (85.7) | 3 (14.3) | 0.78 (0.30-2.06) |  | 55 (82.1) | 12 (17.9) | 0.88 (0.55-1.42) |
| Long-term | 66 (79.5) | 17 (20.5) | 0.87 (0.59-1.3) |  | 29 (78.4) | 8 (21.6) | - 1. (0.46-1.49) |

^1^ Recent immigrants: length of stay < 7 years; Long-term immigrants: length of stay ≥ 7 years.

^2^ Recent immigrants: length of stay < 13 years; Long-term immigrants: length of stay ≥ 13 years.

VCR, vaccination coverage ratio; CI, confidence interval.

^a^ VCR adjusted for sex, age, area of residence, educational level, occupational status, household composition, economic resources, and health services utilization index.

* Reference category for all VCRs presented in the table.
